# The Effect of Plug Rotation Speed on Micro-Structure of Nugget Zone of Friction Plug Repair Welding Joint for 6082 Aluminum Alloy

**DOI:** 10.3390/ma14185287

**Published:** 2021-09-14

**Authors:** Defu Li, Xijing Wang

**Affiliations:** 1 State Key Laboratory of Advanced Processing and Recycling of Non-Ferrous Metals, Lanzhou University of Technology, Lanzhou 730050, China; ldf0301@mail.lzjtu.cn; 2 Faculty of Materials Science and Engineering, Lanzhou University of Technology, Lanzhou 730050, China

**Keywords:** auxiliary heating, friction plug repair welding, preferential orientation, dynamic re-crystallization

## Abstract

This paper carried out the friction plug repair welding of 6082 aluminum alloy keyhole defects by using the method of friction heating between shaft shoulder and base material. In addition, a well-formed friction plug welding joint was obtained at different plug rotation speeds. In order to study the influence mechanism of plug rotation speeds on the microstructure of the weld nugget zone, EBSD technology was used to analyze the grain morphology, grain size and grain boundary characteristics of the weld nugget zone under different rotation speeds of the plug rod. The results show that in the nugget zone, the grain was fine and equated crystals refinement, and there was a preferred orientation. The deformation texture components in the welded nugget zone increased with the plug rotation speed from 1600 to 2000 rpm. However, the grain size first decreased and then increased, while the components in the High-Angle Boundary first increased and then decreased.

## 1. Introduction

Friction stir welding (FSW) does not require filler material during the welding process. If the welding temperature is lower than the melting point of the base metal, metallurgical and crystallization defects can be effectively avoided [1]. Therefore, FSW is recognized as an ideal welding method for aluminum alloy [2], which is widely used in aerospace, rail transportation and other fields [3]. Based on the plastic flow characteristics of materials in the FSW process, the joint can be divided into four zones: weld nugget zone (NZ), thermo-mechanical affected zone (TMAZ), heat affected zone (HAZ) and base metal zone (BM) [4]. Among these, the nugget zone is an “onion” ring structure [5] composed of uniform and finely equated grains, finer than those in other regions [6]. Hofmann [7] conducted underwater multi-channel friction stir machining of 6061 aluminum alloy, and the results showed that the grain size in the weld nugget zone was related to the ambient temperature. Zhao [8] found that the metal in the weld nugget zone was subjected to plastic deformation and flow under the combined action of heat. However, the internal dislocations in the grains increased; in addition, during the welding process these high-density dislocation grains became the core of re-crystallization once again. When the equated grains were refined, the location dislocation density in the weld nugget zone was reduced. Zhang [9] analyzed the 6082 aluminum alloy FSW joint by adopting electron back scattered diffraction (EBSD) technology; results showed that there were both long strip grains and equated grains in the weld nugget zone, that there were small angle grain boundaries in some equated grains, and that the composition of small angle grain boundaries increased. In some regions, the size of the micro-structure of ultra-fine grains was about 2 µm after complete dynamic re-crystallization.

In order to meet the lightweight requirements of high-speed rail transit, 6082 aluminum alloy has been widely used in high-speed rail bodies thanks to its unique performance [10]. Compared to other aluminum alloys, 6082 aluminum alloy belongs to the Al-Mg-Si series alloy, with a tensile strength of 160–320 MPa, elongation of ≥8%, and Mg_2_Si as the strengthening phase. Furthermore, 6082 aluminum alloy is a heat treatment strengthened aluminum alloy, with moderate strength, low density, good corrosion resistance, excellent machinability and weldability, and it can be used for the main body of the high-speed train car body structure [11]. Key holes were unavoidable when friction stir welding was used in aluminum alloy welding [12]. In actual production, the method of adding a lead-out plate was often used to eliminate the FSW keyhole, which was relatively complicated. In order to eliminate keyhole volume defects, Wang [13] took keyhole defects as a research object and repaired the regional volume defects of 2219 aluminum alloy via the solid phase filling +FSW method. The repaired joint strength was no less than 335 MPa. Hao [14] repaired the FSW seam keyhole of 2219 aluminum alloy plate by fusion welding filling + FSW repair welding. The keyhole was filled by manual TIG welding first, then by FSW repair welding, which effectively improved the weld zone structure and changed the structure from cast dendrite to wrought equated crystal, thus improving the repair performance of the keyhole. Zhou [15] repaired the FSW keyhole of 316L stainless steel by filling friction stir welding, and concluded that a hemispherical consumable rod was superior to a conical one, and that the tensile strength reached 92.0% of the weld. JI [16] achieved high-quality repair of volume defects by using the active and passive filling friction stir repair technology of extrusion of metal around defects with needle-free welders, then introduced additional filling metal for passive filling. Reimann [17] used backfill friction stir spot welding to repair through holes in a 6 mm thick 7075-T6 aluminum alloy plate and also obtained defect-free, high-quality joints. 

On the whole, the composite repair method was complicated. Stirring needle extraction technology can eliminate the keyhole [18], but it reduces the effective bearing thickness of the joint. The British Welding Institute invented the friction plug repair welding technology of top forging [19], which can realize the repair of “keyhole” defects in the fusion welding and friction stir welding of high-strength aluminum alloy materials, which were difficult to repair by traditional fusion welding. In order to replace manual TIG welding, this technology was introduced in the United States for repair welding of space external tanks [20]; the parameters were optimized by Lockheed Martin and the Marshall Flight Center. Repair welds with high strength, high fracture toughness and low defect rate were obtained on 2219 and 2195 materials for space external tanks [21]. Beamish [22,23] studied the influence of main parameters such as rotation speed, welding pressure, displacement and plug rod matching angle on joint performance, and obtained the optimized process window on aa6082-T6 aluminum alloy with thicknesses of 10 and 4.7 mm. Dalder [24] carried out top forging friction plug repair welding for the ring seam “keyhole” of a 2219 aluminum alloy pressure vessel with an inner diameter of 1020 mm and a thickness of 38 mm. After removing the micro-defects near the surface by means of mechanical processing, ultrasonic and ultrasonic phased array methods were used for non-destructive testing, and the results showed that the repair welding effect was good. When Michael Lange [25] carried out top forging friction plug repair welding of 2024-T3 aluminum alloy plate with 1/8 inch thickness, he found that the temperature of the base metal near the weld had no obvious fluctuations, and that the joint strength was equivalent to that of the base metal. Du [26] used a 2A14-T6 aluminum alloy conical rod to repair the friction plug welding of 2219-T87 aluminum alloy plate with 10 mm thickness, and also obtained a defect-free joint with a tensile strength up to 312 MPa. Although the friction plug repair welding technology mentioned above could effectively repair the keyhole, it has high requirements on the plug welding equipment due to the high spindle speed, the need to process and remove the redundant plug rods after the repair welding, and the complicated nature of the process. In order to reduce the equipment requirements, Huang [27] used the filled friction stir welding technology to repair the keyhole of 7.8 mm thick 2219 aluminum alloy plate. Through the close contact between the consumable rod, the keyhole, the shaft shoulder and the welder, the friction between the consumable rod, the keyhole, the shaft shoulder and the work piece can produce heat under high speed and high top forging pressure. The plastic deformation and flow of the material occurred at the friction interface and filled the keyhole. The tensile strength and elongation of the joint were 96.0% and 99.0% of the original weld, and the quasi-isometric repair of the defect of the friction stir weld was realized. However, the use of this method was limited to some extent because the consumable rod and the shoulder cannot move relative to one another in the axial position. Ma [28] welded AA7075-T6 aluminum alloy by friction self-impact riveting (F-SPR) and systematically studied the microstructure evolution of aluminum alloy sheeting by the EBSD technique. The results showed that two obvious fine grain regions zones (FGZs) were formed around the rivet and in the rivet cavity with the deformation recrystallization of the rivet and grain. Solid-state bonding of aluminum plates occurred in FGZs. FGZ formation on the outside of the rivet was due to a transition from sliding to sticking at the rivet/sheet interface triggered by dynamic recrystallization (DRX). The FGZ in the rivet cavity was caused by the rotation of the trapped aluminum, which created a sticking affected zone at the trapped aluminum/lower sheet interface and led to DRX. Strain rate gradient in the trapped aluminum drove the further expansion of the sticking affected zone and resulted in grain refinement in a larger span. With the idea of shoulder-assisted heating, the research group carried out the shoulder-assisted friction plug repair welding experiment on 6082 aluminum alloy with split welders [29], which simplified the keyhole volume defect repair process and achieved good results.

Different from friction stir welding with a non-consumable heterogeneous stir needle, friction plug repair welding with a consumable homogeneous tapper plug was adopted; there are essential differences between them in the joint forming mechanism. At present, we are not clear on the mechanism of the effect of the rotation speed of the tapper plug on the micro-structure of the weld nugget zone. Analysis of the grain morphology, grain size and grain boundary characteristics of the weld nugget zone by EBSD technology can provide experimental support for the engineering application of friction plug repair welding technology with applied auxiliary energy field.

## 2. Materials and Methods

**Figure 1:**
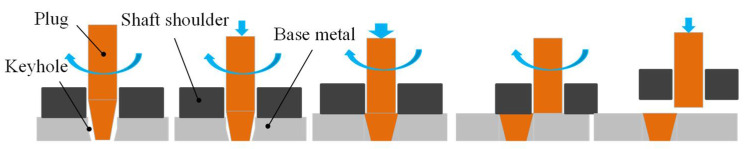
Schematic diagram of the friction plug repair welding process by shaft shoulder auxiliary heating.

Figure 1 shows the experimental principle and process. On the basis of up-forging friction plug repair welding [30], a sleeve shaft-like shoulder was added, and the tapper plug could move axially relative to the shaft shoulder. Before plug welding, the shaft shoulder and the upper surface of the base material contacted and loaded, with the rotation of the shaft shoulder and the friction generating heat and conduction along the base material to achieve the plug hole pre-heating. During plug repair welding, the shaft shoulder and base metal were kept in contact and in friction in order to provide an auxiliary heat source. At the end of up-forging, the shaft shoulder broke the tapper plug laterally along the base metal to form a smooth surface plug repair weld joint.

**Figure 2:**
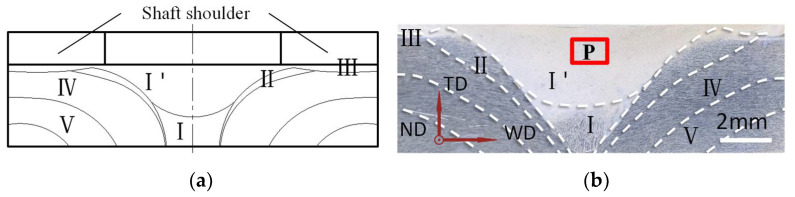
Diagram of a joint’s partition and morphology, and EBSD sampling location. (**a**) Diagram of a joint’s partition. (**b**) When the rotation speed of the plug is 1800 rpm.

The shaft shoulder was made of 9SiCr, and the base metal and tapper plug were made of 6082-T6 aluminum alloy. Table 1 shows the composition. The base metal size was 150 mm × 100 mm × 5 mm, and the diameter of the plug was 10 mm. On the basis of the previous experiment, the angle of the tapper plug and the plug hole was 80°. The tapper plug feed was 6 mm, and experiments were carried out at three different tapper plug rotation speeds: 1600, 1800 and 2000 rpm. Due to the heat input and material deformation, the micro-structure of the joint changed obviously in different areas during friction plug welding. The cross-section morphology of the joint was similar at the three different plug rotation speeds. A complete plug repair welding joint can be divided into six parts: the nugget zone (І ’), populated area (І), combined with surface area (ІІ), shoulder affected zone (ІІІ), thermo-mechanically affected zone (ІV), and heat affected zone (V). Of these, Figure 2 shows the joint cross-section morphology obtained at the joint partition at 1800 rpm.

The EBSD samples were obtained at three rotation speeds in the red area (P), which was at the center of the nugget zone and 1 mm away from the upper surface. Furthermore, the base metal EBSD sample was selected at an appropriate location on the base metal away from the joint. After fine grinding and mechanical polishing, the samples were polished on a Leica EM TIC 3X (Leica, Wetzlar, Germany), an ion grinding and polishing instrument. A field emission scanning electron microscope (Quanta450FEG, FEI Company, Hillsboro, OR, USA) with an EBSD probe and a Channel5 orientation analysis system was used for the EBSD experiment. The sample was tilted 70° and the test voltage was 20 kV. Step size was 1 µm of nugget zone and 5 µm of base metal. (hkl)[uvw] expressed the texture.

## 3. Results and Discussion

### 3.1. Grain Morphology and Grain Boundary Characteristics of the Base Metal

**Figure 3:**
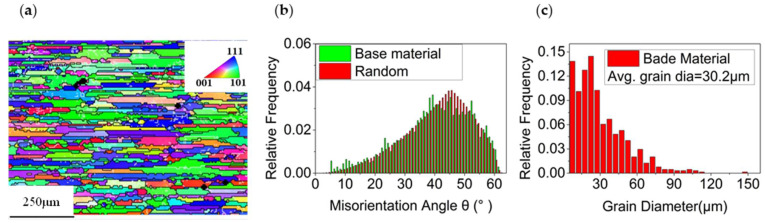
Grain morphology, misorientation angle and grain size of base metal. (**a**) Grain morphology. (**b**) Misorientation angle. (**c**) Grain size.

Figure 3 shows that the base metal was of a typical rolling structure. After aging treatment, the base metal underwent complete re-crystallization, and the grain size was relatively obvious, with an average grain size of 30.2 μm. The orientation difference between grains was close to the free orientation difference, and the grains had no obvious preferred orientation.

### 3.2. Grain Morphology and Grain Boundary Characteristics in the Weld Nugget Zone

**Figure 4:**
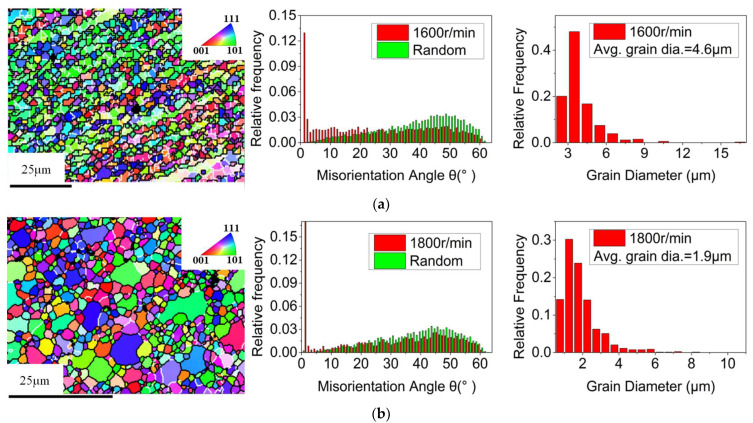

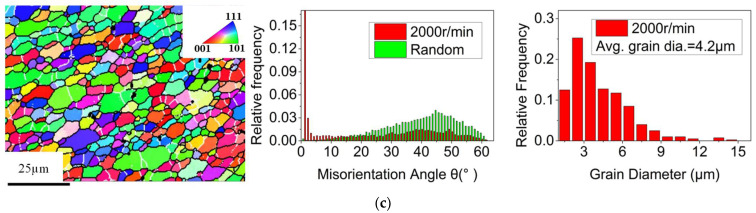
Grain morphology, misorientation angle and grain size of the nugget zone at different plug rotation speeds: (**a**) 1600 rpm, (**b**) 1800 rpm and (**c**) 2000 rpm.

**Figure 5:**
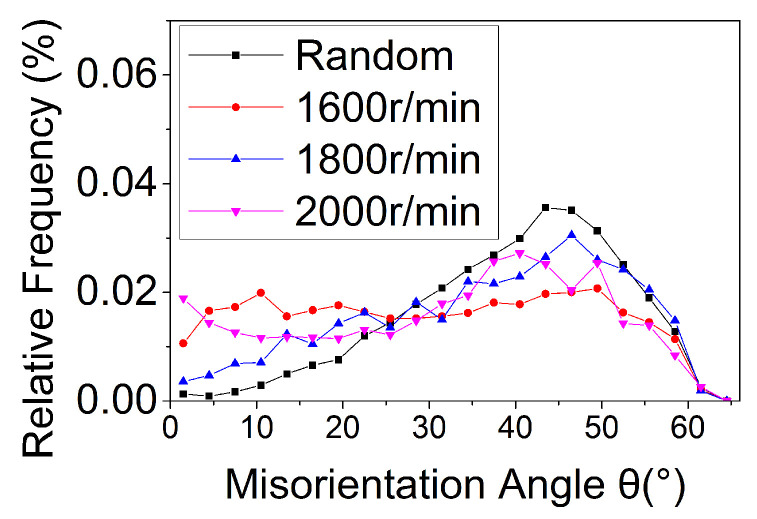
Distribution of misorientation angle at different plug rotation speeds.

Figure 4 shows the grain morphology and orientation difference distribution in the weld nugget zone of the joint under different tapper plug rotation speeds. Among these, white lines show the low-angle grain boundaries, while black lines show the high-angle grain boundaries. Compared with the rolling structure of the base metal, the grain refinement in the weld nugget zone was significant, and the orientation differences among grains deviate from random orientation distribution. Preferred orientation was obvious. Under different plug rotation speeds, Figure 5 shows the comparison of grain orientation difference in the weld nugget zone.

**Figure 6:**
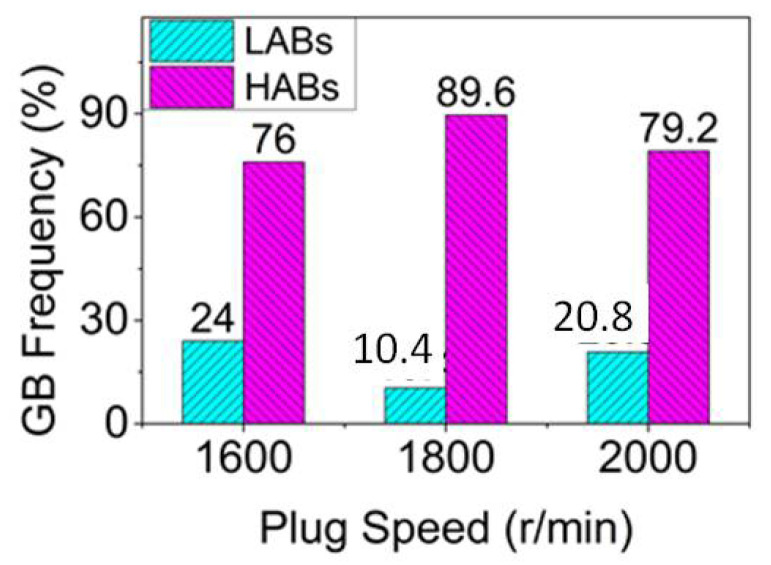
Grain boundary component at different plug rotation speeds.

**Figure 7:**
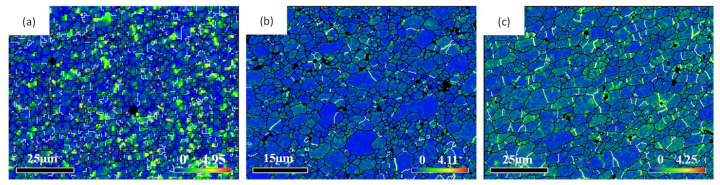
Kernel average misorientation diagram of the weld nugget zone at different plug rotation speeds: (**a**) 1600 rpm, (**b**) 1800 rpm and (**c**) 2000 rpm.

Figure 6 shows that the grain size in the weld nugget zone decreased first and then increased with the increase in the plug rotation speed, while the high-angle boundary component increased first and then decreased. The reason was that with the feeding of the tapper plug, some materials in contact with the plug hole will undergo friction, shear and plastic deformation in the process of friction plug repair welding by the auxiliary heating of the shaft shoulder. Furthermore, the low-angle boundary will increase and form sub-grains due to dislocation proliferation. Under the action of thermal activation energy, the sub-grains combined to form large grains through rotation, the high-angle boundary components increased, dynamic re-crystallization occurred [31], and the grains were refined. This was consistent with the results of grain size and grain boundary composition, which changed when the plug rotation speed increased from 1600 to 1800 rpm. However, when the rotation speed of the plug increased from 1800 to 2000 rpm, the grain size and grain boundary composition changed with the increase in speed. This was because dynamic re-crystallization is a rate-controlled process, and the deformation rate not only affected the nucleation of new grains but also had a great influence on the size of new grains [32]. The increase of the plug rotation speed from 1800 to 2000 rpm caused an increase in deformation temperature, which led to the intensification of thermal vibration and diffusion of atoms in the alloy, the enhancement of grain boundary migration ability, the increase in dynamic re-crystallization nucleation rate, and the coarsening of grains due to the growth of grains. On the other hand, the increase in deformation rate caused by the increase in rotation speed creates a crushing effect on the dynamic recrystallized grains, which increased the location dislocation density of the weld nugget zone, resulting in the increase in low-angle boundary components and the decrease in corresponding high-angle boundary components. This was consistent with the results of stress change and distribution in the weld nugget zone at different plug rotation speeds, as shown in Figure 7.

### 3.3. Texture Types and Components of the Weld Nugget Zone

In order to further study the evolution of grain orientation in the weld nugget zone during friction plug and repair welding, this paper used Channel 5 to calculate the preferred grain orientation in the weld nugget zone at different plug rotating speeds.

**Figure 8:**
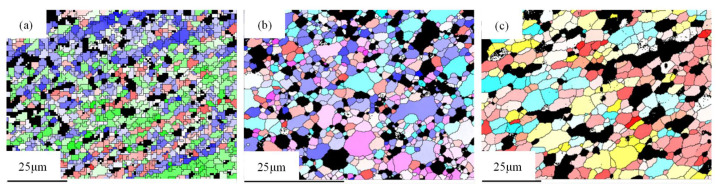
Chromatic aberration and distribution of grain textures at different plug rotating speeds: (**a**) 1600 rpm, (**b**) 1800 rpm and (**c**) 2000 rpm.

Table 2 shows the texture and components of the weld nugget zone at different plug rotating speeds. At 1600 rpm, recrystallized Cube, recrystallized R and (021)[2–12] deformation texture mainly existed in the weld nucleus; at 1800 rpm, recrystallized Cube, (111)[–101] texture, recrystallized Cube and R texture mainly existed; at 2000 rpm, recrystallized Cube and Copper, Goss and (313)[–312] texture mainly existed. Figure 8 shows the color aberration and distribution of grains corresponding to different textures.

In the process of friction plug welding, the tapper plug and the joint material undergo plastic deformation at high temperature, while the plastic deformation of face-centered cubic metal was realized through the motion and interaction of dislocations. In the process of plug welding, the unconsumed cold tapper plug had frictional and shearing effects on the plastic metal formed in the early stage, so the feed of the tapper plug and the rotation of the shaft shoulder would produce stress on the grain in the weld nugget zone. For face-centered cubic aluminum alloys, the final orientation was determined by the stress state of the grains, which led to different textures in the weld nugget zone. When the grains were acted on by the stopper bar and shaft shoulder, the slip plane and the slip direction rotated in a certain direction and finally reached a stable orientation in the face-centered cubic metal, resulting in texture in the weld nugget zone of the joint. The higher the speed of the tapper plug, the higher the grain deformation rate in the weld nugget zone, and the higher the auxiliary heating temperature of the shoulder. On the one hand, high deformation temperature was beneficial to promote the occurrence of dynamic re-crystallization. On the other hand, the high deformation rate also caused the fragmentation of the new nucleated grains in dynamic re-crystallization, resulting in the deformation texture in the weld nugget zone. Therefore, the thermal coupling resulted in the presence of both re-crystallization texture, shear texture and deformation texture. Moreover, the components of re-crystallization and deformation texture increased with the increase in the tapper plug rotating speed.

## 4. Conclusions

- The grain refinement was significant in the weld nugget zone of the friction plug and repair welding joint, and there was an obvious preferred orientation. The grain size in the weld nugget zone decreased first and then increased when the plug rotating speed increased from 1600 to 2000 rpm, while the high-angle boundary component increased first and then decreased.
- At 1600 rpm, recrystallized Cube, recrystallized R and (021)[2–12] deformation texture mainly were found in the nugget zone; at 1800 rpm, Goss, (111)[–101] texture, recrystallized Cube and R texture were found; at 2000 rpm, Copper, Goss, (313)[–312] texture and recrystallized Cube texture were found.
- When the speed of the tapper plug increased, the deformation rate and the temperature of the welding nugget zone increased. The high deformation temperature was beneficial to promote the occurrence of dynamic re-crystallization, while the high deformation rate caused the fragmentation of new nucleated grains. The re-crystallization and the textural components of the weld nugget zone increased with the increase in the plug rotating speed.

## Figures and Tables

**Table 1:** Composition of 6082 aluminum alloy.

Si	Fe	Cu	Mn	Mg	Cr	Zn	Ti	AL
0.89	0.3	0.04	0.58	0.93	0.06	0.04	0.01	BAL

**Table 2:** Texture types and components of the weld nugget zone at different plug rotating speeds.

Color Code	(hkl)[uvw]	1600 rpm	1800 rpm	2000 rpm
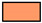	(112)[–1–11]			11.1
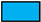	(011)[100]		18.6	18.9
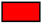	(001)[100]	15.9	13.7	27.6
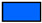	(214)[–1–21]	38.2	30.1	
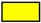	(313)[–312]			22.5
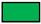	(021)[2–12]	33.8		
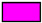	(111)[–101]		19.4	

## Data Availability

The raw/processed data required to reproduce these findings cannot be shared at this time as the data also forms part of an on-going study.
